# Factor structure of the Night Eating Diagnostic Questionnaire (NEDQ) and an evaluation of the diagnostic criteria of the night eating syndrome

**DOI:** 10.1186/s40337-019-0268-9

**Published:** 2019-11-08

**Authors:** Laurence J. Nolan, Allan Geliebter

**Affiliations:** 1 Department of Psychology, Wagner College, 1 Campus Road, Staten Island, NY 10301 USA; 2 Department of Psychiatry, Icahn School of Medicine at Mount Sinai, Mt. Sinai St. Luke’s, New York, NY 10025 USA; 3 Department of Psychology, Touro College and University System, New York, NY 10010 USA

**Keywords:** Night eating syndrome, Sleep disturbance, Depression, Food addiction, Body weight, Mood timing

## Abstract

**Background:**

The Night Eating Diagnostic Questionnaire (NEDQ) is a validated assessment of the night eating syndrome (NES) based on the proposed diagnostic criteria. While past results show that NEDQ is associated with psychopathology and body mass index (BMI), the relationships between the proposed NES diagnostic criteria and psychopathology and BMI have not been investigated. Thus, the relationships between the proposed NES diagnostic criteria and factors previously associated with NES, namely depression, “food addiction” (FA), sleep quality, and BMI were examined. Finally, the NEDQ factor structure was examined for the first time in order to determine whether the NEDQ is assessing NES appropriately.

**Methods:**

The NEDQ, depression, sleep quality, and FA questionnaires were administered to a sample of older community members (*n* = 468) and a student sample (*n* = 254). Principal Components Analysis (PCA) was performed to examine the factor structure of the NEDQ. The relationships between the proposed diagnostic criteria and depression, sleep quality, FA, and BMI were examined using multiple regression.

**Results:**

The proposed NES criteria were highly intercorrelated. PCA revealed a single factor solution for the NEDQ. In the community sample, depression was associated with the presence of five of nine proposed diagnostic criteria while poor sleep quality and FA were associated with the presence of seven and six criteria respectively. In the community sample, only the sleep problems and impairment/distress criteria were positively associated with BMI. In the student sample, fewer criteria were associated with psychopathology, and no criteria were associated with BMI.

**Conclusions:**

These findings support the proposed criteria for diagnosis of NES. All proposed criteria were associated with at least one psychopathology measure. BMI was only associated with the sleep problems and impairment/distress criteria in the community sample. The PCA finding of a single factor indicates that the NEDQ is a cohesive assessment of NES, and that the NEDQ is a good measure of NES criteria.

## Plain English Summary

Night eating syndrome is a proposed eating disorder, in which people may wake up from sleep and often eat to help them go back to sleep. They also eat more than a quarter of their daily food consumption after the evening meal and usually skip breakfast. Night eating has been associated with depression, emotional eating, sleep problems, and food addiction as well as with being overweight or with having obesity (especially as people age). There is some question about the best ways to diagnose night eating. In this paper, we examined the characteristics of a relatively new questionnaire for diagnosis called the Night Eating Diagnostic Questionnaire (NEDQ). We asked 722 women and men to complete a series of questionnaires to determine whether the NEDQ accurately measured all of the proposed criteria of the night eating syndrome, and whether these criteria were linked to depression, sleep problems, food addiction, and body weight. The results showed that the NEDQ assesses all of the night eating criteria. The night eating criteria were linked to higher depression scores, more sleep problems, and more food addiction symptoms, especially in older people. Body weight was associated with night eating only in the older sample.

## Background

Night eating syndrome (NES) is a proposed eating disorder that is characterized by evening hyperphagia and/or nocturnal eating and associated with insomnia and morning anorexia [1, 2], and awakening from sleep to eat [3]. NES prevalence is similar for women and men and is more common in patients with insomnia, obesity, and other psychiatric disorders although most reports are not based on population-based prevalence data [4]. Proposed diagnostic criteria have been developed which require the presence of several of the above features [1]. The core NES criteria include having at least 25% of daily food consumption after the evening meal (evening hyperphagia) and/or the presence of waking in the night to eat (nocturnal eating) [1]. At least 3 of the 5 following criteria should also be present: lack of desire to eat breakfast (morning anorexia), depressed or worse mood in the evening, strong urge to eat between dinner and sleep onset (or during the night), a belief that one must eat in order to fall asleep, and/or sleep onset/maintenance insomnia (sleep problems) [1]. Finally, these symptoms should occur in the absence of the unconscious sleep-related eating disorder, i.e. awareness of eating behavior is present, and in the presence of personal distress and/or impairment associated with the night eating [1]. The Night Eating Diagnostic Questionnaire (NEDQ) was revised to assess NES using these proposed criteria [5, 6]. The NEDQ allows for the assessment of each diagnostic criterion. Additionally, a hierarchical scoring method has been developed to assess the absence of NES (0) and mild (1), moderate (2), and full-syndrome night eating (3) based on the presence of specified criteria [6, 7]. The NEDQ has recently been validated in a university student sample and in a community sample against the Night Eating Questionnaire (NEQ) [7], an older validated measure which produces a continuous score of NES symptom severity [8] but may not, in its current form, directly assess each of the proposed NES criteria [6, 7]. Furthermore, a higher score on the NEDQ has been associated with elevated depression and low self-esteem in adults with obesity [5] and with higher emotional and external eating and poorer sleep quality in university students [9, 10]. NES as reflected by the NEDQ score is associated with elevated depression, poorer sleep quality, elevated “food addiction” (FA) score, in both university students and older community members although the relationships were stronger in the older sample [7]. Most research on NES (often utilizing the NEQ) is limited to examining correlations between symptom severity (e.g., NEQ total score as a continuous variable) and other variables (such as BMI and depression). Other studies compare the characteristics of those who have met the criterion for NES (e.g., NEQ score ≥ 25) to those who have not. These studies do not allow for the assessment of the inclusion of the proposed criteria or for the examination of the relationship of the proposed clinical diagnostic criteria to psychopathology. There has been increased interest in NES in the past 10 years and the call for additional support for the proposed diagnostic criteria remains highly relevant [11] given that little published work has examined the proposed NES diagnostic criteria. Finally, the factor structure of the NEDQ has not yet been reported. Analysis of the factor structure would allow for the assessment of the degree to which all of the proposed diagnostic criteria are relevant and might suggest further refinements to the NEDQ.

The present study had several purposes: the first was to determine to what extent the key proposed diagnostic criteria of NES as measured by the NEDQ load onto one or more components using principal component analysis. The second was to examine to what degree the presence of each of the proposed diagnostic criteria is associated with depression and sleep quality (given the association between NES and both depression and sleep quality) and with FA given its relationship with a variety of eating disorders [12]. Because we previously showed that NEDQ score (based on number of symptoms) is associated with these psychopathological factors [7], our aim was to see how the individual criteria were related to these psychopathological factors. These analyses would allow us to determine the relationship between proposed diagnostic criteria and psychopathology as well as provide a measure of the cohesiveness of the proposed NES diagnostic criteria. Finally, we also examined which of the NES criteria were associated with elevated BMI. Elevated BMI has sometimes been associated with NES, particularly in clinical samples [4, 8]. In previous studies [9, 13] and in the present community (but not student) sample, NEDQ score was positively correlated with BMI [7].

## Method

### Participants

Participants included community members (*n* = 468) who participated via an online survey (Qualtrics, Provo, UT) and university students who were tested in a university laboratory (*n* = 254). Both samples of participants were predominantly comprised of persons who identified as white (approximately 80%). One quarter of community members volunteered to participate via the university staff email distribution list while the remainder were recruited and paid for their time via Qualtrics sample service. Student participants were compensated with research experience credits, which they were required to accumulate as part of an introductory psychology course. Sample characteristics are reported in Table 1. In the process of validating the NEDQ, the relationship between depression, sleep quality, FA, BMI, and NEDQ in these samples has been published [7]. The relationship of these psychopathology variables using the NEQ as a measure of NES symptom severity has also been published for this student sample and a subset of this community sample [14].

**Table 1:** Sample characteristics (Mean ± SEM)

	Student	Community	Statistic	*p*
*N*	254	468		
	Mean ± SEM		t (df)	
Age/years	18.7 ± 0.1	42.9 ± 0.6	−27.9 (715)	<.001
BMI	24.5 ± 0.3	28.0 ± 0.3	−7.7 (711)	<.001
SDS	37.8 ± 0.6	40.8 ± 0.5	−3.9 (720)	<.001
PSQI	6.5 ± 0.2	7.3 ± 0.2	−2.8 (718)	0.006
YFAS	1.9 ± 0.1	2.2 ± 0.1	−2.4 (720)	0.017
	%		χ^2^(df)	
Sex (Women)	63.1	55.8		
BMI group			61.5 (3)	<.001
Obese	9.8	32.2		
Overweight	26.0	30.9		
Normal	60.2	34.4		
Underweight	3.9	2.4		
NEDQ			13.6 (3)	0.003
None	79.5	73.9		
Mild	11.8	9.4		
Moderate	6.3	7.3		
Full	2.4	9.4		

*BMI* Body mass index, *SDS* Self-report Depression Scale, *PSQI* Pittsburgh Sleep Quality Index, *YFAS* Yale Food Addiction Scale, *NEDQ* Night Eating Diagnostic Questionnaire, *df* degrees of freedom

### Measures

Night Eating. The Night Eating Diagnostic Questionnaire (NEDQ) [5] includes 22 questions (often yes/no) and is designed to assess the presence of each of the proposed criteria for diagnosis of NES [1, 6]. The criteria assessed by the NEDQ and the hierarchical scoring method (not used in the present study) are presented in Table 2. Internal consistency is not assessed because the NEDQ is a symptom checklist. The first version of the NEDQ [6], was based on Stunkard et al. [2] and was subsequently revised to reflect the NES criteria proposed by Allison et al. [1]. Convergent validity with the NEQ has been demonstrated [7]. The NEDQ has been utilized in a variety of populations. Based on the NEDQ, the prevalence of NES in adults with obesity ranged from 8.3% in patients treated with gastric bypass surgery [15] to 14.0% for those in a weight loss program [5]. In studies of convenience samples of university students, the prevalence based on the NEDQ was reported to range from 1.2% [16] and 2.4% [14] to as high as 5.7% [9]. In a study of Turkish university students, 9.5% met the criteria for NES based on the NEDQ [17]. The only study of a national convenience sample of adults using the NEDQ found a prevalence of 9.4% [7].

**Table 2:** Proposed NES Diagnostic Criteria as Assessed by the NEDQ

A. One or both of the following.	
1) At least 25% of food intake is consumed after the evening meal.	
2) At least two nocturnal eating episodes per week.	
B. Awareness and recall of evening and nocturnal eating episodes are present.	
C. At least three of the following.	
1) Lack of desire to eat in the morning and/or breakfast is omitted four or more times per week.	
2) Presence of a strong urge to eat between dinner and sleep onset and/or during the night.	
3) Sleep maintenance and/or onset insomnia are present four or more times per week.	
4) Presence of a belief that one must eat in order to initiate or return to sleep.	
5) Mood is frequently depressed or mood is worse in evening.	
D. The disorder is associated with significant distress and/or impairment in functioning.	
E. Maintenance of disordered eating for at least 3 months.	
F. The disorder is not secondary to substance abuse or dependence, medical disorder, medication, or another psychiatric disorder.)	
Experimental Hierarchical Scoring	
0. Non-NE = normal (does not meet any criteria category below)	
1. N = mild night eater has at least 1 criterion from A (but does not meet criteria for NE or NES)	
2. NE = moderate night eater has at least 1 criterion from A plus ≥3 of 5 qualifiers from criteria C (but does not meet criteria for NES)	
3. NES = full syndrome night eater has at least 1 criterion from A plus ≥3 of 5 qualifiers from criteria C plus D and E	

#### Sleep quality

The Pittsburgh Sleep Quality Index (PSQI) is composed of 19 Likert-type items to assess sleep habits during the previous month [18]. High scores (total scores can range from 0 to 21) indicate poorer sleep quality. For the present study, internal consistency (Cronbach’s alpha) was 0.67 for the student sample and 0.84 for the community sample. The PSQI has seven subscales which measure overall subjective sleep quality, sleep disturbance, sleep duration, sleep efficiency, sleep latency, dysfunction in the daytime due to sleepiness, and frequency of drug use (over-the-counter and prescription) to promote sleep.

#### Depression

The Zung Self-report Depression Scale (SDS) consists of 20 Likert-type questions (half positively phrased, half reverse-scored negatively phrased) to which the participant responds on a 4-point Likert-type scale [19]. The total score ranges from 20 to 80 with a score above 50 common in depressed persons [20]. In the present study, internal consistency (Cronbach’s alpha) was good with values of 0.85 for the student sample and 0.86 for the community sample. In all analyses, the SDS was entered as a continuous variable.

#### Food addiction

The Yale Food Addiction Scale (YFAS) was designed to evaluate “addiction” toward foods according to the DSM-IV criteria for substance dependence [21] and is widely used. It is composed of 25 Likert-type questions. In the present study, internal consistence (Kuder-Richardson’s alpha) was 0.86 for the student sample and 0.96 for the community sample. The YFAS is scored by counting the number of proposed diagnostic criteria that are met (0–7). In the analyses presented below, the YFAS was entered as a continuous variable (number of symptoms).

### Procedure

The Wagner College Human Experimental Review Board approved all experimental procedures, and all participants provided informed consent. Potential participants were asked to take part in a study examining “eating and sleeping habits” [7]. For the student sample, questionnaires were administered in alternating order in paper booklets with demographic questions presented after the psychological measures. Students were tested in groups, and each student completed the questionnaires while seated alone at a table. The height and weight of each student were measured using a stadiometer and digital scale, respectively, after the questionnaire booklet was completed. For the community (online) sample, questionnaire administration was similar, except heights and weights were self-reported after completion of the psychological questionnaires. In order to determine eligibility for the study and to ensure gender balance, the participants who were recruited by Qualtrics were first asked their age and gender. At the end of the test session (whether online or in laboratory), the participants were debriefed as to the purpose of the study.

### Analysis

All statistical analyses were performed using IBM SPSS (version 24) unless otherwise stated. In order to determine which proposed diagnostic criteria were present, responses to the NEDQ items were scored in a binary fashion to determine the presence (score = 1) or absence (score = 0) of the NES criteria for the past 3 or more months. When performing principal components analysis (PCA) with dichotomous variables, there is a risk of bias toward more factors if it is performed using Pearson product-moment correlation coefficients [22]. The tetrachoric correlation coefficient was developed to calculate the correlation between dichotomous measures of theoretically underlying continuous variables [23, 24]. Thus, a matrix of tetrachoric correlations for the proposed NES diagnostic criteria (generated using STATA version 13.1) was used for the PCA procedure. Parallel analysis [25] was used to confirm the number of factors extracted using the online Parallel Analysis Engine [26]. Parallel analysis is the preferred method of selecting factors which compares the observed eigenvalues with those obtained from Monte-Carlo simulated matrix created from random data of the same sample size, thereby reducing the chances of selecting factors that have an eigenvalue above 1 by chance [27, 28]. In parallel analysis, components are retained when the obtained eigenvalue is greater than expected values from 100 randomly generated correlation matrices.

Multiple regression was performed to examine to what degree each of the proposed diagnostic criteria was associated with psychopathology (i.e., depression and FA as well as sleep quality). Multiple regression was also used to examine which of these criteria was associated with BMI. For BMI, age was entered into the model as a predictor along with the proposed diagnostic criteria because it is an associated factor with the relationship between BMI and NES symptom severity [29]. Because the relationships between NDEQ scores and psychopathology and BMI measures were stronger in the community sample than they were in students [7], separate analyses were performed for each sample when examining how the presence of NES criteria predicted psychopathology and BMI. Standardized beta coefficients are presented for all analyses. Collinearity was low (VIF values < 1.2) for all regression analyses.

## Results

### Symptom frequency

Of the proposed diagnostic criteria, depressed mood later in the day was the most common in the entire sample; 51.9% reported that their mood was lower in the afternoon or evening. Morning anorexia was reported by 50.0% while 46.0% experienced urge to eat between evening meal and sleep, 38.4% reported sleep problems, 18.0% reported evening hyperphagia, 10.5% reported nocturnal eating, and 5.5% reported feeling that they needed to eat in order to sleep. 9.6% reported awareness of their night eating and 17.5% reported feeling distressed or impaired by it.

### Principal component analysis

**Fig. 1:**
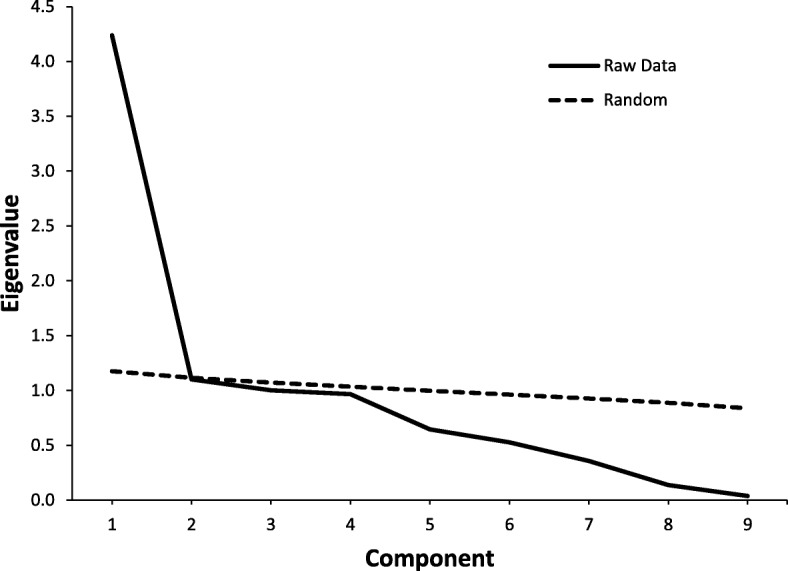
Scree plot for principal components analysis and parallel analysis for the NES criteria. The random line represents parallel analysis eigenvalues for randomly generated data for the same sample size

The tetrachoric correlation matrix for the proposed diagnostic criteria was similar for the samples of students and community members, so they were pooled for the PCA. The results revealed significant positive correlations among the proposed NES diagnostic criteria (see Table 3). The Kaiser-Meyer-Olkin measure of sampling adequacy was .76, and the Bartlett test of sphericity was highly significant (X^2^_28_ = 4241.7, *p* <. 001), indicating acceptability to perform the PCA. The PCA revealed that there were three components with eigenvalues above 1 (and one more just below 1), with the first contributing 47.1% and the second and third contributing 12.2 and 11.1% of the variance, respectively. However, the shape of the scree plot suggested only one significant component, which was confirmed by parallel analysis (see Fig. 1).

**Table 3:** Tetrachoric correlation coefficient (rho) matrix for the proposed night eating syndrome diagnostic criteria (*N* = 722)

Criterion	Evening Hyperphagia	Nocturnal Ingestion	Morning Anorexia	Sleep Problems	Evening Urge to Eat	Eat to Sleep	Lower Mood in Evening	Aware
Nocturnal Ingestion	.46***							
Morning Anorexia	.32***	.23**						
Sleep Problems	.22**	.44***	.24***					
Evening Urge to Eat	.46***	.44***	.16*	.19**				
Must Eat to Sleep	.45***	.91***	.12	.30**	.51***			
Lower Mood	.07	.22**	.09	.16**	.27***	.26**		
Aware	.37***	.92***	.13	.37***	.36***	.82***	.19*	
Distress/ Impaired	.46***	.62***	.26***	.21**	.59***	.65***	.18*	.46***

Note: statistical significance for tetrachoric coefficients is based on standard error

*p < .05, **p < .01, ***p < .001

### Multiple regression analyses for proposed NES diagnostic criteria

#### Depression

The regression model predicting depression score from all proposed diagnostic criteria was highly statistically significant in the community sample, F(9, 458) = 28.34, *p* = .000 (adjusted R-squared = .35), and in the student sample, F(9, 240) = 10.02, *p* = .000 (adjusted R-squared = .25). In the community sample, the proposed diagnostic criteria associated with higher depression scores included evening hyperphagia, lower mood later in day, sleep problems, morning anorexia, and distress (see Table 4), but in the student sample, only morning anorexia, lower mood later in day, and sleep problems were associated with depression score (see Table 5).

**Table 4:** Regression of each psychopathology measure and BMI on NES diagnostic criteria for the community sample (β = standardized beta)

	Criterion Variable											
	SDS			PSQI			YFAS			BMI		
	Β	t	*p*	β	t	*p*	β	t	*p*	β	t	*p*
Evening Hyperphagia	.11	2.69	.007	.11	3.04	.003	.14	3.15	.002	−.04	−0.82	.411
Nocturnal Eating	.06	0.93	.355	−.02	− 0.45	.656	.15	2.37	.018	.06	0.77	.441
Depressed Mood	.15	3.91	.000	.15	4.45	.000	.14	3.37	.001	−.01	−0.19	.854
Must Eat to Sleep	.06	1.15	.251	.09	2.11	.035	.03	0.50	.616	−.07	−1.13	.258
Evening Urge to Eat	.01	0.23	.820	−.02	−0.46	.644	.16	3.74	.000	.05	0.89	.372
Sleep Problems	.33	8.37	.000	.53	15.22	.000	.13	3.19	.002	.11	2.23	.026
Morning Anorexia	.15	3.89	.000	.11	3.31	.001	.03	0.73	.466	.00	−0.04	.965
Awareness	.00	0.07	.947	.11	2.35	.019	−.07	−1.35	.178	−.04	−0.66	.511
Distress/Impairment	.18	3.95	.000	.10	2.65	.008	.21	4.48	.000	.18	3.14	.002

*SDS* Zung Self-rating Depression Scale, *PSQI* Pittsburgh Sleep Quality Index, *YFAS* Yale Food Addiction Scale, *BMI* Body mass index

**Table 5:** Regression of each psychopathology measure and BMI on diagnostic criteria for the student sample (β = standardized beta)

	Criterion Variable											
	SDS			PSQI			YFAS			BMI		
	β	t	*p*	β	t	*p*	β	t	*p*	β	t	*p*
Evening Hyperphagia	.05	0.81	.805	−.02	−0.38	.703	.06	1.01	.313	−.09	−1.35	.178
Nocturnal Eating	−.10	−1.38	.168	−.11	−1.58	.116	−.09	−1.11	.018	.02	0.27	.785
Depressed Mood	.16	2.77	.006	.13	2.30	.022	.08	1.29	.200	−.02	−0.28	.779
Must Eat to Sleep	.07	1.25	.214	.01	0.13	.893	.01	0.08	.936	−.04	−0.55	.584
Evening Urge to Eat	.10	1.73	.085	.06	1.06	.292	.16	2.38	.018	.05	0.73	.469
Sleep Problems	.29	5.11	.000	.16	8.33	.000	.10	1.56	.119	.02	0.25	.805
Morning Anorexia	.21	3.61	.000	.15	2.68	.008	.06	1.02	.308	.07	1.01	.313
Awareness	.05	0.71	.482	.00	−0.06	.956	−.02	−0.27	.784	−.03	−0.31	.755
Distress/Impairment	.19	3.21	.001	.01	0.24	.808	.18	2.79	.006	.03	0.51	.612

*SDS* Zung Self-rating Depression Scale, *PSQI* Pittsburgh Sleep Quality Index, *YFAS* Yale Food Addiction Scale, *BMI* Body mass index

#### Sleep quality

The regression model predicting sleep quality from all proposed diagnostic criteria was highly statistically significant in the community sample, F(9,456) = 52.69, *p* = .000 (adjusted R-squared = .50), and in the student sample, F(9,240) = 10.75, *p* = .000 (adjusted R-squared = .26). Poor sleep quality was associated in both samples with morning anorexia, low mood in evening, and sleep problems. However, evening hyperphagia, need to eat in order to sleep, awareness of night eating, and distress/impairment were associated with poor sleep quality only in the community group (see Tables 4 and 5 for regression coefficients).

#### Food addiction

The regression model predicting FA from all proposed diagnostic criteria was highly statistically significant in the community sample, F(9,458) = 22.09, *p* = .000 (adjusted R-squared = .29) and in the student sample, F(9,240) = 3.90, *p* = .000 (adjusted R-squared = .10). In the community sample, all of the proposed diagnostic criteria except the belief that one must eat to sleep, morning anorexia, and awareness of night eating were associated with more FA symptoms (see Table 4). In the student sample, nocturnal eating, urge to eat between dinner and sleep time, and distress/impairment were associated with more FA symptoms (see Table 5).

#### Body mass index

The model predicting BMI from all proposed diagnostic criteria was statistically significant in the community sample, F(9,449) = 2.54, *p* = .008 (adjusted R-squared = .05; see Table 4). Only sleep problems and distress/impairment were associated with higher BMI. The inclusion of age did not alter the outcome. The model was not statistically significant in the student sample, F(9,240) = 0.43, *p* = .917 (adjusted R-squared = −.02; see Table 5). Given the very restricted age range in the student sample, age was not entered into the regression model.

## Discussion

The results of the PCA indicated that the NEDQ assesses a single construct, which supports the proposed diagnostic criteria as providing a cohesive diagnosis for NES. The factor structure confirms that the NEDQ assesses the NES criteria as intended, and that revisions to the NEDQ are not necessary at this time.

The analysis of proposed diagnostic criteria showed that in the community sample, five of the proposed diagnostic criteria were associated with elevated depression, six with FA, and seven with lower sleep quality. The criteria associated with all psychopathology included evening hyperphagia, depressed mood later in the day, and sleep problems (see Table 6 for a summary). Thus, the positive relationships between NES (based on NES severity by NEQ and by NEDQ score) and these psychological factors, which have been previously reported, are also present for most of the proposed diagnostic criteria. Poor sleep quality was associated with most of the criteria, which is not surprising given that one of the salient features of NES is nocturnal food consumption, which would be disruptive of sleep. Furthermore, poor sleep quality is associated with several psychopathologies [30, 31]. Allison et al. [32], using the NEQ, reported support for the core diagnostic features of late eating and nocturnal ingestion as well as for sleep disturbances such as initial insomnia and nocturnal awakenings, using item response theory analysis. However, they reported that morning anorexia did not differentiate those with night eating. The current findings show that morning anorexia is correlated with the other criteria (although with fewer of them than the other criteria) and associated with poor sleep quality and higher depression scores when the other criteria were present in the multiple regression analysis. These results suggest that morning anorexia should remain as one of the five optional NES diagnostic criteria as proposed [1]. Finally, the distress/impairment criterion is associated with all psychological measures and BMI in the community sample.

**Table 6:** Summary of the relationships between NES diagnostic criteria and psychological and BMI measures. Single check indicates relationship for community sample only. Double check mark indicates relationship for both student and community samples. Criteria are organized by proposed diagnostic requirements [1]

NES Diagnostic Criteria	Depression	Sleep Quality	“Food Addiction”	BMI
At least one of these:				
Evening Hyperphagia	✓	✓	✓	
Nocturnal Eating			✓✓	
At least three of these:				
Depressed Mood in Evening	✓✓	✓✓	✓	
Sleep Problems	✓✓	✓✓	✓	✓
Morning Anorexia	✓✓	✓✓		
Need to Eat to Sleep		✓		
Urge to Eat in Evening			✓	
Required:				
Awareness		✓		
Impaired/Distressed	✓✓	✓	✓✓	✓

*NES* Night eating syndrome, *BMI* Body mass index

In university students, FA was associated with evening hyperphagia and craving to eat in the evening. Depression and poor sleep quality were associated with morning anorexia, depressed mood later in the day, and sleep problems. Furthermore, in students, psychopathology was less associated with the core features of NES and the distress/impairment criterion. For each criterion, students had fewer associations with psychopathology; there were no associations in students that were not present in the older community sample. Thus, students may exhibit a milder, incomplete form of NES consistent with onset appearing in late adolescence and early adulthood [4]. It is also likely that some of the differences between the students and community samples were due to the lower frequency of BMI values ≥25 in the student sample. It has been suggested that NES may contribute to increased weight over time [33] which may be why BMI is only related to the proposed NES diagnostic criteria in the community adult sample.

In this study, FA was associated with the presence of the core criteria of NES, in addition to several others, as well as psychological distress/impairment from night eating. FA has been associated with eating disorders and subclinical problem eating as well as higher BMI [12]. However, the concept of FA is controversial. Advocates of FA suggest that some foods (e.g., energy-dense “processed” highly palatable foods) generate addiction-like behaviors in those who ingest them [20]. Some critics prefer an alternative description that focuses on the behavior (i.e., “eating addiction”) and suggest there is little evidence that food is an addicting substance, and that overeating may be a form of habitual food “abuse” [34] or represent a possible food use disorder [35]. In addition, there may be a lack of evidence to conclude that FA is a distinct entity that explains overeating [36]. Nonetheless, there has been growing interest in FA in the scientific community [37].

Only the sleep problems and distress/impairment from night eating criteria were associated with elevated BMI (and only in the older community sample). This finding is consistent with previous research, which shows that poor sleep quality [38] and short sleep duration [39] are each positively correlated with BMI. These results suggest that nocturnal eating and other core features of NES may not contribute to higher BMI. However, BMI was associated with the presence of subjective problems due to night eating itself. Elevated BMI has been associated with NES symptom severity as measured by NEQ score [13] and NEDQ score [7], but NES is not consistently associated with BMI in the general population and rarely in students. While BMI has been weakly associated with NEQ score in college students in Germany [40], this association has not been found in US student samples [9, 14, 41]. NES was prevalent but not related to obesity in a population-based study of Australian adolescents [42]. Although a weak significant correlation with BMI has been found (using the NEQ) in general population adults in Germany [29] and the US [14], usually the relationship is stronger and more consistently found in clinical populations [8]. NEDQ score has also been weakly, but significantly positively associated with BMI in a community-based sample, but the relationship was not found in students [9, 16]. For self-recorded daily food intake, those with NES were found to consume a daily energy similar to control participants but to exhibit a shift of that energy to later in the day [43]. Thus, the relationship between NES and BMI may have more to do with when energy is consumed than the total amount. The NES pattern of food intake is manifested similarly in those of normal weight and in those with obesity [33].

Our interpretations of these results have some limitations. The data were obtained from convenience samples. Although we were able to compare different age groups, the conclusions are limited by the cross-sectional design. Only a longitudinal study would allow for the examination of developmental processes in regard to NES, psychopathology, and BMI. The samples tested allow for good generalization to older members of the population and to university students, with the possible exception of populations with more diverse ethnicity. Because the community sample did not include persons below 21 years of age, the results have limited generalizability to young adults who are not enrolled in higher education. Data from students were obtained in person with anthropometric measures for BMI, whereas data from community members were obtained via online surveys with self-reported weight and height to compute BMI. However, care was taken to ensure the reliability of online data, and self-reported weight and height are highly correlated with measured height and weight and BMI [44]. In addition, the prevalence of overweight and obesity in the community group was consistent with published rates in the general population [45]. Another limitation is the lack of an inclusion of a binge eating measure to exclude those who might meet the criteria for binge eating disorder (BED). While BED and NES are distinct entities [46], up to 25% of those diagnosed with NES using the NEQ may also meet the criterion for BED [1]. The incidence of BED in those diagnosed with NES using the NEDQ is unknown. Persons with NES, like those with BED, may report a loss of control during their nocturnal eating episodes, but the amount they eat does not appear to resemble a binge in size [46]. Furthermore, unlike those with BED, the loss of control over eating appears to be restricted to the nighttime for those with NES [46]. Finally, these findings are limited by the lack of inclusion of variables related to NES such as self-esteem, anxiety, and stress [2, 5].

## Conclusions

The results of this study show that the NEDQ has a single factor structure, which supports the validity of the proposed diagnostic criteria for NES. Furthermore, the associated psychopathology was linked to each of the proposed diagnostic criteria in a community sample (more so than in the student sample) suggesting that psychopathology is not only linked to overall symptom severity but to the presence of specific clinical features of NES. BMI was not associated with core features of NES; however, BMI was associated with sleep problems and night eating related distress/impairment. The results support the use of the NEDQ as an accurate and cohesive diagnostic tool for NES.

## Data Availability

The data set is available from the corresponding author upon reasonable request.

## Abbreviations

BED: Binge eating disorder
BMI: Body mass index
FA: Food addiction
NEDQ: Night Eating Diagnostic Questionnaire
NEQ: Night Eating Questionnaire
NES: Night eating syndrome
PCA: Principal components analysis
PSQI: Pittsburgh Sleep Quality Index
VIF: Variance inflation factors
YFAS: Yale Food Addiction Scale
